# Maternal and neonatal outcomes among Chinese women meeting NICE but not IADPSG criteria for gestational diabetes mellitus: A retrospective cohort study

**DOI:** 10.7555/JBR.39.20250350

**Published:** 2026-07-28

**Authors:** Haijuan Yu, Yuan Qin, Runlian Tao, Yuanyuan Chen, Li Yuan, Lizhou Sun

**Affiliations:** 1 Department of Obstetrics, the First Affiliated Hospital of Nanjing Medical University, Nanjing, Jiangsu 210029, China; 2 Department of Obstetrics, Nanjing Jiangning Hospital of Chinese Medicine, Nanjing, Jiangsu 211100, China; 3 Affiliated Jiangning Hospital of Chinese Medicine, China Pharmaceutical University, Nanjing, Jiangsu 211198, China; 4 Department of Biochemistry and Molecular Biology, Jiangsu Key Laboratory of Molecular Targets and Intervention for Metabolic Diseases, Nanjing Medical University, Nanjing, Jiangsu 211166, China

**Keywords:** gestational diabetes mellitus, IADPSG, NICE, incidence, adverse pregnancy outcomes

## Abstract

In China, gestational diabetes mellitus (GDM) is typically diagnosed using the International Association of Diabetes and Pregnancy Study Groups (IADPSG) criteria. This study retrospectively analyzed 19152 pregnant women who underwent an oral glucose tolerance test between 2015 and 2021, comparing the IADPSG and the National Institute for Health and Care Excellence (NICE) criteria. GDM prevalence was 20.39% (IADPSG) and 23.67% (NICE, *P* < 0.01), with a moderate diagnostic agreement (κ = 0.59; *P* < 0.01). Compared with women having normal glucose tolerance (NGT group), women diagnosed with GDM by either the IADPSG (GDM-I) or NICE (GDM-N) criteria had significantly higher risks of adverse outcomes (including gestational hypertension, preeclampsia, preterm delivery, macrosomia, low birth weight, and neonatal jaundice). However, those diagnosed by NICE but not by IADPSG (GDM-NI) showed no significant increase in adverse maternal or neonatal outcomes except for neonatal jaundice. Furthermore, the GDM-NI group had significantly lower rates of several adverse outcomes compared with the GDM-I group. These findings suggest that, under current clinical management where women diagnosed by IADPSG received treatment while those diagnosed only by NICE did not, the IADPSG criteria classified fewer women as having GDM but identified a group with a higher observed risk of several adverse pregnancy outcomes than did the NICE criteria.

## Introduction

Gestational diabetes mellitus (GDM), defined as any degree of glucose intolerance with onset or first recognition during pregnancy, is increasing in prevalence worldwide, driven in part by rising obesity rates^[1]^. According to the 2021 International Diabetes Federation report, approximately 16.7% (21.1 million) of live births globally were affected by maternal hyperglycemia, with GDM accounting for 80.3% of cases, pre-existing diabetes for 10.6%, and first-diagnosed diabetes during pregnancy (including type 1 and type 2 diabetes) for 9.1%^[2]^. Untreated GDM poses significant risks to both the mother and baby, increasing the risks of maternal and neonatal adverse outcomes such as preeclampsia, preterm delivery, cesarean delivery, macrosomia, shoulder dystocia, and neonatal hyperbilirubinemia^[3–4]^. However, early diagnosis through standardized screening allows for prompt intervention with dietary control, physical activity advice, or even insulin therapy if needed, thereby markedly improving pregnancy outcomes^[5]^.

The reported prevalence of GDM varies widely among populations, ranging from 5% to 25%^[6–7]^. This variability stems not only from geographical and ethnic factors but also from the screening methods and diagnostic criteria applied. Currently, there is no universal consensus on the definition of GDM, leading to significant variations in diagnostic thresholds, screening timing, and testing methodologies. Historically, although GDM-specific criteria such as the Carpenter–Coustan and National Diabetes Data Group standards were widely used before 2008, they were primarily designed to predict the mother's long-term risk of developing postpartum diabetes rather than immediate adverse perinatal outcomes. In 2010, the International Association of Diabetes and Pregnancy Study Groups (IADPSG) established new diagnostic criteria for GDM based on glucose thresholds associated with an odds ratio of 1.75 for adverse pregnancy outcomes, as identified in the Hyperglycemia and Adverse Pregnancy Outcomes (HAPO) study. These criteria recommend a 75-g oral glucose tolerance test (OGTT) at 24–28 weeks of gestation, with thresholds of fasting plasma glucose (FPG) ≥ 5.1 mmol/L, 1-h glucose ≥ 10.0 mmol/L, or 2-h glucose ≥ 8.5 mmol/L (***Supplementary Table 1***)^[8]^. Although multiple studies have shown that the IADPSG criteria identify more GDM cases than the two-step approach^[9–10]^, Duran *et al*^[10]^ demonstrated that the application of the IADPSG criteria was associated with improved pregnancy outcomes at a lower cost compared with prior diagnostic criteria. Consequently, the IADPSG criteria have since been widely adopted by major organizations, including the World Health Organization^[11]^, the American Diabetes Association (ADA)^[12]^, the Australian Diabetes in Pregnancy Society^[13]^, and the European Board and College of Obstetrics and Gynecology^[14]^. In China, both the 2014 "Guidelines for Diagnosis and Treatment of Diabetes in Pregnancy" (published in Chinese)^[15]^ and the 2022 "Guidelines for Diagnosis and Treatment of Hyperglycemia in Pregnancy"^[16]^ also endorse the IADPSG diagnostic approach.

However, the IADPSG criteria's lower FPG cutoff has raised concerns regarding potential overdiagnosis, increased resource allocation, and increased medicalization of pregnancy^[17]^. In contrast, the UK's National Institute for Health and Care Excellence (NICE) developed alternative criteria in 2015, which feature a higher fasting threshold (≥ 5.6 mmol/L), a lower 2-h cutoff (≥ 7.8 mmol/L), and the exclusion of the 1-h measurement, based on comprehensive health economic modeling of immediate pregnancy complications (***Supplementary Table 1***). Several studies have found that the NICE criteria result in a lower prevalence of GDM than the IADPSG criteria^[18–20]^ and are cost-effective^[20]^. This divergence in diagnostic approaches reflects ongoing debate about optimal GDM screening strategies and threshold determinations, and to date, no studies have specifically compared these diagnostic approaches in Chinese populations.

In the present study, we aimed to compare the performance of the IADPSG and NICE criteria for diagnosing GDM and to assess pregnancy outcomes in women diagnosed by NICE but not by IADPSG in a Chinese cohort.

## Subjects and methods

### Study population

This retrospective cohort study included 19152 women with singleton pregnancies who received complete prenatal care and delivered at the First Affiliated Hospital of Nanjing Medical University between January 2015 and December 2021. Inclusion criteria were: (1) singleton live birth after 28 weeks' gestation; and (2) completion of 75-g OGTT at 24–28 weeks of gestation. We excluded pregnancies with any of the following conditions: pre-gestational diabetes mellitus (defined as diabetes diagnosed prior to pregnancy or overt diabetes in pregnancy^[21]^—FPG ≥ 7.0 mmol/L, 2-h plasma glucose ≥ 11.1 mmol/L on a 75-g OGTT, random plasma glucose ≥ 11.1 mmol/L, or glycated hemoglobin ≥ 6.5%), multiple gestation, fetal demise (stillbirth or miscarriage), pre-existing chronic medical conditions, severe pregnancy complications, or incomplete clinical records.

All procedures in this study were approved by the ethics committee of the First Affiliated Hospital of Nanjing Medical University (Approval No. 2021-SR-055). Informed consent was waived due to the retrospective nature of the study.

### Information collection

Maternal and neonatal data were obtained from electronic medical records. Maternal parameters included: (1) demographic data (age, body weight at OGTT, and height); (2) metabolic parameters (75-g OGTT values at 24–28 weeks of gestation); and (3) maternal and neonatal outcomes (gestational hypertension, preeclampsia, cesarean delivery, preterm delivery, and macrosomia). Neonatal outcomes included fetal distress and neonatal jaundice. Body mass index (BMI) was calculated by dividing body weight at OGTT in kilograms by the square of height in meters.

### Diagnostic definition of GDM by IADPSG and NICE criteria

All participants completed a standardized 75-g OGTT between 24–28 weeks of gestation. As shown in ***Supplementary Table 1***, women were defined as having GDM by IADPSG criteria if their FPG was ≥ 5.1 mmol/L, and/or 1-h OGTT was ≥ 10.0 mmol/L, and/or 2-h OGTT was ≥ 8.5 mmol/L. Women were defined as having GDM by NICE criteria if their FPG was ≥ 5.6 mmol/L and/or 2-h OGTT was ≥ 7.8 mmol/L. Notably, while the NICE guidelines recommend a selective, risk-factor-based screening strategy in clinical practice, all participants in this retrospective cohort routinely underwent universal 75-g OGTT screening. Therefore, this study specifically evaluated and compared the NICE diagnostic glucose thresholds against the IADPSG criteria within a universally tested population, rather than simulating the full NICE screening strategy.

All women diagnosed with GDM by IADPSG criteria received intensive dietary and lifestyle counseling plus instructions for self-monitoring of capillary glucose, in accordance with Chinese guidelines derived from IADPSG. Insulin therapy was initiated if FPG exceeded 5.3 mmol/L or 2-h postprandial glucose exceeded 6.7 mmol/L despite optimized interventions. However, individual-level data on treatment uptake, adherence, and specific insulin usage were unavailable in this registry database. ***Table 1*** presents the baseline characteristics of the study population classified by IADPSG criteria. The enrolled subjects exhibited typical clinical features of GDM.

**Table 1:** Baseline characteristics of the study population by IADPSG criteria

Characteristics	Total (*n* = 19152)	Non-GDM (*n* = 15246)	GDM (*n* = 3906)	*P*
Age at OGTT (years, mean ± SD）	30.96 ± 4.65	30.82 ± 4.61	31.5 ± 4.76	< 0.01
BMI at OGTT (kg/m^2^, mean ± SD)	27.51 ± 3.72	27.44 ± 3.68	27.8 ± 3.87	< 0.01
FPG (mmol/L, mean ± SD)	4.59 ± 0.85	4.39 ± 0.35	5.37 ± 1.51	< 0.01
1-h OGTT (mmol/L, mean ± SD)	7.64 ± 1.18	7.64 ± 1.18	9.22 ± 1.93	< 0.01
2-h OGTT (mmol/L, mean ± SD)	6.69 ± 1.50	6.35 ± 1.10	8.03 ± 2.02	< 0.01
Birth weight (g, mean ± SD)	3391.94 ± 469.85	3385.23 ± 452.76	3418.11 ± 530.59	< 0.01
Gestational hypertension [*n* (%)]	611 (3.19)	407 (2.67)	204 (5.22)	< 0.01
Preeclampsia [*n* (%)]	673 (3.51)	451 (2.96)	222 (5.68)	< 0.01
Preterm delivery [*n* (%)]	944 (4.93)	635 (4.17)	309 (7.91)	< 0.01
Fetal distress [*n* (%)]	391 (2.04)	304 (1.99)	87 (2.22)	0.359
APH [*n* (%)]	112 (0.58)	86 (0.56)	26 (0.67)	0.460
PPH [*n* (%)]	1856 (9.69)	1481 (9.71)	375 (9.6)	0.831
Cesarean delivery [*n* (%)]	7436 (38.83)	5841 (38.31)	1595 (40.83)	0.004
Macrosomia [*n* (%)]	1421 (7.42)	1020 (6.69)	401 (10.27)	< 0.01
LBW [*n* (%)]	567 (2.96)	384 (2.52)	183 (4.69)	< 0.01
Neonatal jaundice [*n* (%)]	7724 (40.33)	5571 (36.54)	2153 (55.12)	< 0.01
Data are expressed as mean ± standard deviation (SD), or numbers (percentages). Differences between the two groups were tested by the chi-square test for categorical variables, Student's *t*-test for normally distributed continuous variables, and the Mann–Whitney *U* test for non-normally distributed variables. Abbreviations: APH, antepartum hemorrhage; BMI, body mass index; FPG, fasting plasma glucose; GDM, gestational diabetes mellitus; LBW, low birth weight; OGTT, oral glucose tolerance test; PPH, postpartum hemorrhage.				

### Definition of maternal and neonatal outcomes

We compared ten adverse maternal and neonatal outcomes: (1) Gestational hypertension (blood pressure > 140/90 mmHg after the 20th week of pregnancy without proteinuria); (2) Preeclampsia (blood pressure > 140/90 mmHg after the 20th week of pregnancy and proteinuria > 300 mg per 24 hours); (3) Antepartum hemorrhage (any vaginal blood loss after the 24th week of gestation); (4) Postpartum hemorrhage (≥ 500 mL of blood loss within 24 h in vaginal delivery or ≥ 1000 mL in cesarean delivery); (5) Preterm delivery (< 37 weeks of gestation); (6) Fetal distress (a combination of symptoms that endanger the health and life of the fetus in utero due to acute or chronic hypoxia); (7) Cesarean delivery; (8) Macrosomia (birth weight > 4000 g); (9) Low birth weight (birth weight < 2500 g); and (10) Neonatal jaundice (requiring phototherapy).

### Statistical analysis

Statistical analyses were performed using IBM SPSS Statistics for Windows, Version 27.0 (IBM Corp., Armonk, NY, USA). Continuous variables were expressed as mean ± standard deviation. For comparisons among multiple groups, one-way ANOVA followed by Bonferroni post hoc test was used for normally distributed data, and the Kruskal–Wallis test followed by Dunn's test with Bonferroni correction was used for skewed data. Categorical variables were expressed as *n* (%) and were compared using Pearson's chi-square test with Bonferroni's post hoc test. Unadjusted and adjusted logistic regression analyses were employed to evaluate odds ratios (ORs), along with their 95% confidence intervals (CIs). Adjusted models included age and BMI as covariates. Diagnostic agreement between the criteria was evaluated with Cohen's kappa coefficient (κ: 0.8–1.0, very good; 0.6–0.8, good; 0.4–0.6, moderate). Statistical significance was set at *P* < 0.05.

## Results

### Prevalence of GDM by the IADPSG and NICE criteria

**Figure 1:**
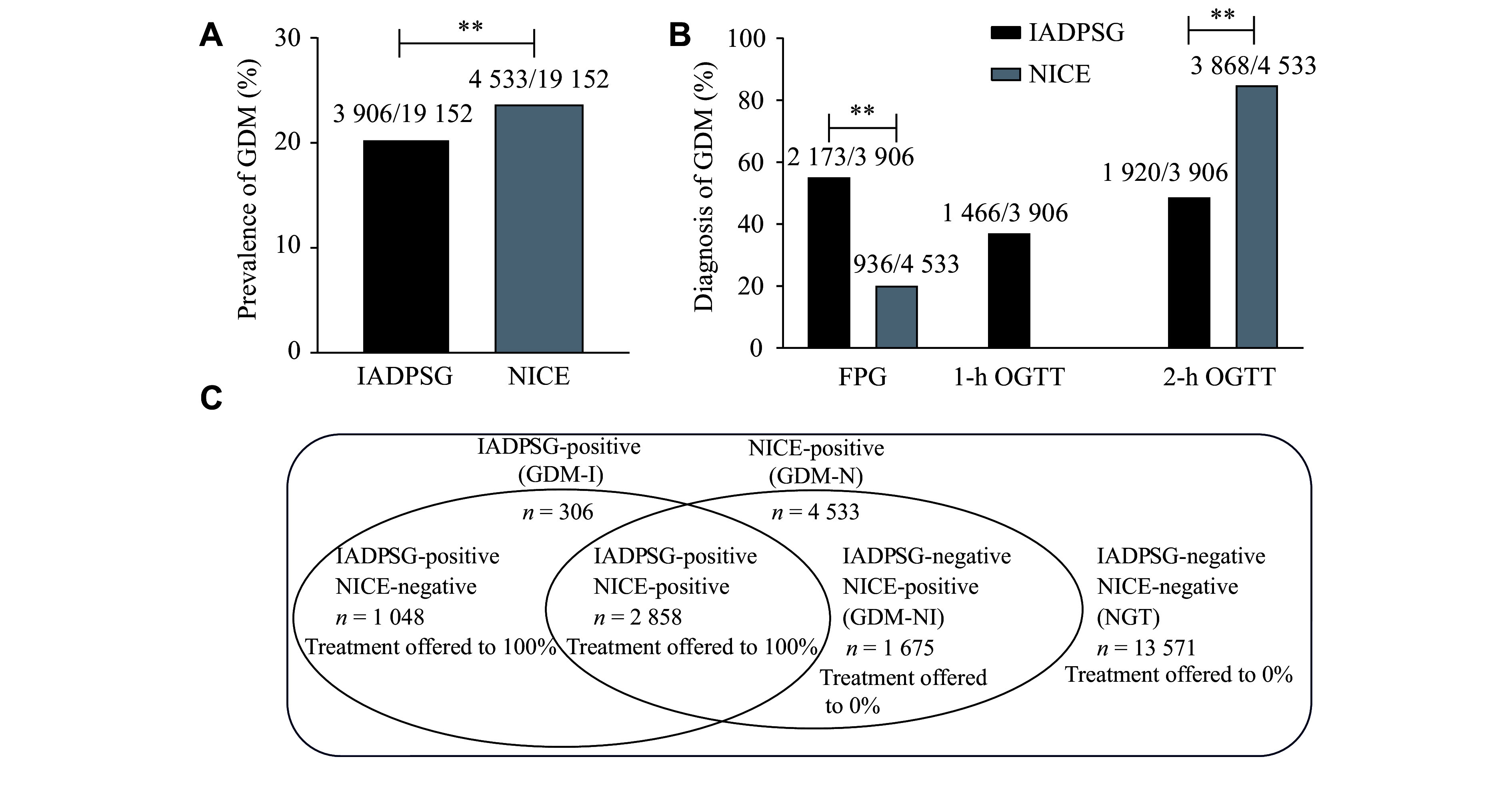
Prevalence of GDM by IADPSG and NICE criteria. A: Percentage of patients diagnosed with GDM according to IADPSG and NICE criteria. B: Classification of pregnant women according to GDM diagnosis. C: Percentage of patients with GDM diagnosed by at least one plasma glucose level of OGTT. ^**^P < 0.01 by chi-square test. Abbreviations: FPG, fasting plasma glucose level; IADPSG, International Association of Diabetes and Pregnancy Study Groups; NGT, normal glucose tolerance; NICE, National Institute for Health and Care Excellence; OGTT, oral glucose tolerance test.

As shown in ***Fig. 1A***, among the 19152 pregnant women, the prevalence of GDM was 20.39% (*n* = 3906) using the IADPSG criteria, which increased significantly to 23.67% (*n* = 4533) using the NICE criteria (*P* < 0.001), indicating a notable difference in detection rates. Agreement between the criteria was moderate (κ = 0.59, *P* < 0.01).

Diagnostic concordance analysis (***Fig. 1C***) revealed that 14.92% (*n* = 2858) met both IADPSG and NICE criteria, 5.47% (*n* = 1048) were identified exclusively by IADPSG, and 8.75% (*n* = 1675) exclusively by NICE. The majority of cases (70.86%, *n* = 13571) were classified as non-GDM by both criteria.

Analysis of OGTT values (***Fig. 1B***) revealed that elevated FPG identified 55.63% (*n* = 2173) of GDM cases by IADPSG, but only 20.65% (*n* = 936) by NICE (*P* < 0.01). Conversely, the 2-h OGTT plasma glucose detected more GDM cases using NICE (85.33%, *n* = 3868) than IADPSG (49.16%, *n* = 1920, *P* < 0.01). The 1-h OGTT, included only in IADPSG, contributed to 37.53% (*n* = 1466) of GDM diagnoses.

When considering any single OGTT value (***Table 2***), under IADPSG, 39.25% were diagnosed solely by elevated FPG, 6.68% by abnormal 1-h glucose, and 37.69% by abnormal 2-h glucose; 7.09% met two abnormal thresholds, and 9.29% met all three. Under NICE, 14.67% were diagnosed by FPG alone, 79.35% by elevated 2-h glucose, and 5.98% met both thresholds.

**Table 2:** Distribution of GDM women based on IADPSG or NICE criteria

Plasma glucose	IADPSG (*n* = 3906)	NICE (*n* = 4533)
Only FPG	1533 (39.25)	665 (14.67)
Only 1-h OGTT	261 (6.68)	–
Only 2-h OGTT	1472 (37.69)	3597 (79.35)
Any two abnormal values	277 (7.09)	271 (5.98)
All three abnormal values	363 (9.29)	–
Values are presented as *n* (%). "–" indicates data not available.Abbreviations: FPG, fasting plasma glucose; IADPSG, International Association of Diabetes and Pregnancy Study Groups; NICE, National Institute for Health and Care Excellence; OGTT, oral glucose tolerance test.		

### Evaluation of maternal variables, maternal and neonatal outcomes

Women diagnosed with GDM by IADPSG received glycemic management, whereas those in the NICE^+^/IADPSG^−^ (GDM-NI) group did not receive therapy. Women were classified into four subgroups: NGT (IADPSG^−^/NICE^−^), GDM-I (IADPSG^+^), GDM-N (NICE^+^), and GDM-NI (NICE^+^/IADPSG^−^) (***Fig. 1B*** and ***Table 3***).

**Table 3:** The classification of the study population according to the OGTT results and GDM diagnostic criteria

Groups	Definitions	Plasma glucose level (mmol/L)		
		Fasting	1-h OGTT	2-h OGTT
NGT^*^	Non-GDM (IADPSG-negative, NICE-negative)	< 5.1	< 10.0	< 7.8
GDM-I^&^	GDM according to IADPSG criteria	≥ 5.1	≥ 10.0	≥ 8.5
GDM-N^&^	GDM according to NICE criteria	≥ 5.6	–	≥ 7.8
GDM-NI^*^	GDM according to NICE criteria, but not IADPSG criteria	< 5.1	< 10.0	7.8–8.4
To convert glucose value from mmol/L to mg/dL, multiply by 18. "–" indicates data not available.^*^Pregnant women whose glucose values were all below the diagnostic thresholds for both IADPSG and NICE criteria were assigned to this group.^&^Pregnant women with any glucose value that met the thresholds were assigned to this group.Abbreviations: GDM, gestational diabetes mellitus; GDM-I, GDM diagnosed by IADPSG criteria; GDM-N, GDM diagnosed by NICE criteria; GDM-NI, GDM diagnosed by NICE but not by IADPSG; NGT, normal glucose tolerance; IADPSG, International Association of Diabetes and Pregnancy Study Groups; NICE, National Institute for Health and Care Excellence; OGTT, oral glucose tolerance test.				

***Table 4*** presents the maternal and demographic characteristics and pregnancy outcomes stratified by the GDM classification used in this analysis. In total, 13571 pregnant women had no GDM (*i.e.*, normal glucose tolerance by both IADPSG and NICE criteria, IADPSG^−^/NICE^−^, the NGT group), 3906 met criteria for GDM based on the IADPSG criteria (IADPSG^+^, the GDM-I group), 4533 met criteria for GDM based on the NICE criteria (NICE^+^, the GDM-N group), and 1675 were diagnosed with GDM based on the NICE criteria but not the IADPSG criteria (NICE^+^/IADPSG^−^, GDM-NI). As expected, pregnant women with GDM diagnosed by any of the criteria were older and had higher BMI, OGTT plasma glucose, and newborn birth weight than those with normal glucose tolerance (***Table 4***). There was no significant difference in newborn birth weight between the GDM-NI and NGT groups.

**Table 4:** Maternal and neonatal characteristics stratified by OGTT-based diagnostic classification

Characteristics	NGT (*n* = 13571)	GDM-I (*n* = 3906)^*^	GDM-N (*n* = 4533)^*^	GDM-NI (*n* = 1675)	*P*
Age at OGTT (years)	30.78 ± 4.62^A^	31.5 ± 4.76^B^	31.45 ± 4.71^B^	31.21 ± 4.51^B^	< 0.001
BMI at OGTT (kg/m^2^)	27.42± 3.66^A^	27.8 ± 3.87^B^	27.68 ± 3.85^B^	27.62 ± 3.89^B^	< 0.001
FPG (mmol/L)	4.39 ± 0.35^A^	5.37 ± 1.51^B^	5.08 ± 1.48^B^	4.45 ± 0.34^B^	< 0.001
1-h OGTT (mmol/L)	7.51 ± 1.16^A^	9.25 ± 1.95^B^	9.25 ± 1.61^B^	8.71 ± 0.74^B^	< 0.001
2-h OGTT (mmol/L)	6.13 ± 0.96^A^	8.03 ± 2.02^B^	8.47 ± 1.54^B^	8.11 ± 0.20^B^	< 0.001
Birth weight (g)	3383.41 ± 449.84^A^	3418.11 ± 530.59^B^	3407.09 ± 506.69^B^	3400.02 ± 475.65^AB^	0.001
Gestational hypertension [*n* (%)]	354 (2.61)^A^	204 (5.22)^B^	208 (4.59)^BC^	53 (3.16)^AC^	< 0.001
Preeclampsia [*n* (%)]	390 (2.87)^A^	222 (5.68)^B^	208 (4.59)^BC^	61 (3.64)^AC^	< 0.001
Preterm delivery [*n* (%)]	553 (4.07)^A^	309 (7.91)^B^	308 (6.79)^B^	82 (4.90)^A^	< 0.001
Fetal distress [*n* (%)]	273 (2.01)	87 (2.22)	89 (1.96)	31 (1.85)	0.371
APH [*n* (%)]	72 (0.53)	26 (0.67)	33 (0.73)	14 (0.84)	0.400
PPH [*n* (%)]	1314 (9.68)	375 (9.60)	450 (9.93)	167 (9.97)	0.731
Cesarean delivery [*n* (%)]	5204 (38.35)^A^	1595 (40.83)^B^	1811 (39.95)^AB^	637 (38.03)^AB^	0.034
Macrosomia [*n* (%)]	893 (6.58)^A^	401 (10.27)^B^	407 (8.98)^BC^	127 (7.58)^AC^	< 0.001
LBW [*n* (%)]	334 (2.46)^A^	183 (4.69)^B^	185 (4.08)^BC^	50 (2.99)^AC^	< 0.001
Neonatal jaundice [*n* (%)]	4890 (36.03)^A^	2153 (55.12)^B^	2352 (51.89)^C^	681 (40.66)^D^	< 0.001
Data are expressed as mean ± standard deviation (SD), or numbers (percentages). Continuous variables were compared using one-way ANOVA with Bonferroni post hoc test for normally distributed data, or Kruskal–Wallis test with Dunn's test for skewed data. Categorical variables were compared using the chi-square test with Bonferroni's post hoc test. Values in the same row with different superscript letters differ significantly (*P* < 0.05). A value with superscripts 'AB' is not significantly different from values with superscript 'A' or 'B'. *Some women were in both the GDM-I and GDM-N groups. Abbreviations: APH, antepartum hemorrhage; BMI, body mass index; FPG, fasting plasma glucose; GDM, gestational diabetes mellitus; GDM-I, GDM diagnosed by IADPSG criteria; GDM-N, GDM diagnosed by NICE criteria; GDM-NI, GDM diagnosed by NICE but not by IADPSG; IADPSG, International Association of Diabetes and Pregnancy Study Groups; LBW, low birth weight; NICE, National Institute for Health and Care Excellence; NGT, normal glucose tolerance; OGTT, oral glucose tolerance test; PPH, postpartum hemorrhage.					

As shown in ***Table 4***, women diagnosed with GDM by IADPSG (GDM-I) or NICE (GDM-N) had significantly increased rates of gestational hypertension (5.22% and 4.59% *vs.* 2.61%), preeclampsia (5.68% and 4.59% *vs.* 2.87%), preterm delivery (7.91% and 6.78% *vs.* 4.07%), macrosomia (10.27% and 8.98% *vs.* 6.58%), low birth weight (LBW, 4.69% and 4.08% *vs.* 2.46%), and neonatal jaundice (55.12% and 51.89% *vs.* 36.03%) compared with the NGT group. Moreover, the GDM-I group had a significantly higher cesarean delivery rate than the NGT group (40.83% *vs.* 38.35%), while the GDM-N group showed no significant difference compared with the NGT group (39.95% *vs.* 38.35%). Further analysis revealed that, compared with the GDM-I group, the GDM-N group exhibited decreased rates across gestational hypertension, preeclampsia, preterm delivery, fetal distress, cesarean delivery, macrosomia, LBW, and neonatal jaundice, with only neonatal jaundice demonstrating a statistically significant decrease (51.89% *vs.* 55.12%). These findings suggest that, within the current clinical management strategy, the IADPSG criteria identify women at higher risk of adverse pregnancy outcomes.

After adjustment for maternal age and BMI, the increased risks of adverse outcomes in the GDM-I and GDM-N groups remained significant, although the magnitude of association was slightly attenuated (***Table 5***). Specifically, the GDM-NI group did not show significantly increased risks for most adverse outcomes after adjustment, except for neonatal jaundice (adjusted OR = 1.22, 95% CI: 1.10–1.35).

**Table 5:** Odds ratios for adverse pregnancy outcomes by OGTT diagnostic classification

Outcomes	OR type	NGT group (*n* = 13571)	GDM-I^*^ (*n* = 3906)	GDM-N^*^ (*n* = 4533)	GDM-NI (*n* = 1675)
Gestational hypertension	Unadjusted	1.00 (Ref.)	2.06 (1.73–2.45)	1.80 (1.51–2.14)	1.22 (0.91–1.64)
	Adjusted	1.00	1.95 (1.63–2.33)	1.72 (1.44 –2.05)	1.17 (0.87–1.58)
Preeclampsia	Unadjusted	1.00	2.04 (1.72–2.41)	1.63 (1.37–1.93)	1.28 (0.97–1.68)
	Adjusted	1.00	1.94 (1.64–2.31)	1.57 (1.32–1.86)	1.23 (0.93–1.63)
Preterm delivery	Unadjusted	1.00	2.02 (1.75–2.34)	1.72 (1.49–1.98)	1.21 (0.96–1.54)
	Adjusted	1.00	2.02 (1.75–2.34)	1.72 (1.49–1.98)	1.21 (0.96–1.54)
Fetal distress	Unadjusted	1.00	1.11 (0.87–1.42)	0.98 (0.77–1.24)	0.92 (0.63–1.34)
	Adjusted	1.00	1.15 (0.90–1.46)	1.01 (0.79–1.28)	0.94 (0.65–1.37)
APH	Unadjusted	1.00	1.26 (0.80–1.97)	1.38 (0.91–2.08)	1.58 (0.89–2.81)
	Adjusted	1.00	1.24 (0.79–1.95)	1.36 (0.90–2.06)	1.57 (0.88–2.79)
PPH	Unadjusted	1.00	0.99 (0.88–1.12)	1.03 (0.92–1.15)	1.03 (0.87–1.22)
	Adjusted	1.00	1.00 (0.89–1.13)	1.04 (0.93–1.16)	1.04 (0.88–1.23)
Cesarean delivery	Unadjusted	1.00	1.11 (1.03–1.19)	1.07 (0.99–1.15)	0.99 (0.89–1.10)
	Adjusted	1.00	1.11 (1.03–1.19)	1.07 (1.00–1.14)	0.99 (0.89–1.09)
Macrosomia	Unadjusted	1.00	1.62 (1.44–1.84)	1.40 (1.24–1.58)	1.17 (0.96–1.41)
	Adjusted	1.00	1.59 (1.40–1.80)	1.38 (1.22–1.56)	1.15 (0.95–1.39)
LBW	Unadjusted	1.00	1.95 (1.62–2.34)	1.69 (1.40–2.03)	1.22 (0.90–1.65)
	Adjusted	1.00	1.96 (1.63–2.35)	1.69 (1.41–2.03)	1.22 (0.90–1.65)
Neonatal jaundice	Unadjusted	1.00	2.18 (2.03–2.34)	1.91 (1.79–2.05)	1.22 (1.10–1.35)
	Adjusted	1.00	2.20 (2.04–2.36)	1.92 (1.80–2.06)	1.22 (1.10–1.35)
Data are presented as odds ratios (ORs) with 95% confidence intervals (CIs). Unadjusted and adjusted logistic regression analyses were employed to evaluate ORs, along with their 95% CIs. Adjusted models included age and BMI at OGTT as covariates, because other variables were unavailable, such as parity, previous GDM, family history of diabetes, gestational weight gain, smoking, *in vitro* fertilization, and assisted reproduction. ^*^Some women were in both the GDM-I and GDM-N groups. Abbreviations: APH, antepartum hemorrhage; BMI, body mass index; GDM, gestational diabetes mellitus; GDM-I, GDM diagnosed by IADPSG criteria; GDM-N, GDM diagnosed by NICE criteria; GDM-NI, GDM diagnosed by NICE but not by IADPSG; IADPSG, International Association of Diabetes and Pregnancy Study Groups; LBW, low birth weight; NICE, National Institute for Health and Care Excellence; NGT, normal glucose tolerance; OGTT, oral glucose tolerance test; PPH, postpartum hemorrhage.					

Further analysis revealed that, compared with the NGT group, women in the GDM-NI group showed a significantly higher incidence of neonatal jaundice (40.66% *vs.* 36.03%), whereas no significant differences were observed in any of the other outcomes. However, when compared with the GDM-I group, the GDM-NI group had significantly lower rates of gestational hypertension (3.16% *vs.* 5.22%), preeclampsia (3.64% *vs.* 5.68%), preterm delivery (4.90% *vs.* 7.91%), macrosomia (7.58% *vs.* 10.27%), low birth weight (2.99% *vs.* 4.69%), and neonatal jaundice (40.66% *vs.* 55.12%). Additionally, there were no significant differences among the four groups in terms of fetal distress, antepartum hemorrhage (APH), or postpartum hemorrhage (PPH).

### Association between fasting/post-load plasma glucose levels and pregnancy outcomes

Numerous studies have shown that only elevated FPG significantly increases the risk of adverse pregnancy outcomes^[22–23]^. To explore why the GDM-NI group exhibited a lower rate of adverse pregnancy outcomes, participants were categorized into distinct subgroups within their respective diagnostic frameworks (IADPSG and NICE) to examine the specific impacts of fasting versus post-load hyperglycemia. Specifically, under the IADPSG framework, participants were identified as IADPSG-only FPG elevation or IADPSG-only post-load elevation; similarly, within the NICE framework, they were classified as NICE-only FPG elevation or NICE-only post-load elevation. It should be noted that these subgroups are not mutually exclusive across the two frameworks, and separate logistic regression analyses were conducted within each independent diagnostic system using NGT as the common reference.

As expected, compared with the NGT group, all GDM subgroups showed significantly higher risks for several key adverse pregnancy outcomes. Within the IADPSG framework, the IADPSG-only FPG subgroup exhibited a higher risk for adverse outcomes compared to the IADPSG-only post-load subgroup. Similarly, within the NICE framework, the NICE-only FPG subgroup demonstrated a more pronounced risk than the NICE-only post-load subgroup (***Table 6***). Further analysis revealed that the IADPSG-only FPG group and NICE-only FPG group had a significant increase in gestational hypertension (4.96% *vs.* 3.75% and 4.36% *vs.* 3.95%), preeclampsia (6.91% *vs.* 3.69% and 5.26% *vs.* 4.14%), preterm delivery (8.61% *vs.* 6.35% and 9.77% *vs.* 5.98%), macrosomia (10.76% *vs.* 8.37% and 10.08% *vs.* 8.23%), and low birth weight (LBW, 4.83% *vs.* 4.10% and 5.71% *vs.* 3.67%) compared with their respective post-load groups (IADPSG-only post-load or NICE-only post-load), with the exception of neonatal jaundice (40.51% *vs.* 65.78% and 39.25% *vs.* 53.68%). After adjustment for maternal age and BMI at OGTT, isolated fasting hyperglycemia remained significantly associated with several adverse pregnancy outcomes within both diagnostic frameworks (***Table 7***). The adjusted ORs for neonatal jaundice in the IADPSG-only post-load group remained markedly elevated, suggesting that maternal post-load glucose spikes might independently influence neonatal bilirubin pathways rather than being a mere reflection of maternal age and BMI. These findings indicate that GDM with isolated fasting hyperglycemia was associated with higher risks of several adverse pregnancy outcomes after adjustment for maternal age and BMI.

**Table 6:** Association of different GDM diagnostic indices with pregnancy outcomes within independent diagnostic frameworks

Outcomes	NGT (*n* = 13571)	IADPSG-only FPG (*n* = 1533)	IADPSG-only post-load (*n* = 1733)	NICE-only FPG (*n* = 665)	NICE-only post-load (*n* = 3597)	*P*
Gestational hypertension [*n* (%)]	354 (2.61)^A^	76 (4.96)^B^	65 (3.75)^AB^	29 (4.36)^AB^	142 (3.95)^B^	< 0.001
Preeclampsia [*n* (%)]	390 (2.87)^A^	106 (6.91)^B^	64 (3.69)^AC^	35 (5.26)^BC^	149 (4.14)^C^	< 0.001
Preterm delivery [*n* (%)]	553 (4.07)^A^	132 (8.61)^BC^	110 (6.35)^CD^	65 (9.77)^B^	215 (5.98)^D^	< 0.001
Fetal distress [*n* (%)]	273 (2.01)	42 (2.74)	30 (1.73)	18 (2.71)	63 (1.75)	0.110
APH [*n* (%)]	72 (0.53)	10 (0.65)	9 (0.52)	5 (0.75)	25 (0.70)	0.740
PPH [*n* (%)]	1314 (9.68)	143 (9.33)	164 (9.46)	68 (10.23)	350 (9.73)	0.970
Cesarean delivery [*n* (%)]	5204 (38.35)	609 (39.73)	715 (41.26)	272 (40.9)	1420 (39.48)	0.100
Macrosomia [*n* (%)]	893 (6.58)^A^	165 (10.76)^B^	145 (8.37)^ABC^	67 (10.08)^BC^	296 (8.23)^C^	< 0.001
LBW [*n* (%)]	334 (2.46)^A^	74 (4.83)^B^	71 (4.1)^B^	38 (5.71)^B^	132 (3.67)^B^	< 0.001
Neonatal jaundice [*n* (%)]	4890 (36.03)^A^	621 (40.51)^B^	1140 (65.78)^C^	261 (39.25)^AB^	1931 (53.68)^D^	< 0.001
Data are expressed as numbers (percentages). Categorical variables were compared using the chi-square test with Bonferroni's post hoc test. Values in the same row with different superscript letters differ significantly (*P* < 0.05). A value with superscripts 'AB' is not significantly different from values with superscript 'A' or 'B'. Subgroups within this table are defined based on individual diagnostic criteria frameworks and are not mutually exclusive. Abbreviations: APH, antepartum hemorrhage; IADPSG-only FPG, gestational diabetes mellitus (GDM) women with only elevated fasting plasma glucose diagnosed by IADPSG; IADPSG-only-post-load, GDM women with elevated only post-load hyperglycemia (1 h and/or 2 h) diagnosed by IADPSG; LBW, low birth weight; NGT, normal glucose tolerance; NICE-only FPG, GDM women with elevated only fasting plasma glucose diagnosed by NICE; NICE-only post-load, GDM women with elevated only post-load hyperglycemia (2 h) diagnosed by NICE; PPH, postpartum hemorrhage; Ref., reference;						

**Table 7:** Odds ratios for adverse pregnancy outcomes analyzed separately by the IADPSG and NICE criteria

Outcomes	OR type	NGT (*n* = 13571)	IADPSG-only FPG (*n* = 1533)	IADPSG-only post-load (*n* = 1733)	NICE-only FPG (*n* = 665)	NICE-only post-load (*n* = 3597)
Gestational hypertension	Unadjusted	1.00 (Ref.)	1.95 (1.51–2.51)	1.46 (1.11–1.91)	1.70 (1.16–2.51)	1.54 (1.26–1.87)
	Adjusted	1.00	1.81 (1.40–2.34)	1.45 (1.11–1.90)	1.57 (1.06–2.32)	1.50 (1.23–1.83)
Preeclampsia	Unadjusted	1.00	2.51 (2.01–3.13)	1.30 (0.99–1.70)	1.88 (1.32–2.68)	1.46 (1.21–1.77)
	Adjusted	1.00	2.35 (1.88–2.94)	1.31 (1.00–1.72)	1.74 (1.21–2.49)	1.44 (1.19–1.75)
Preterm delivery	Unadjusted	1.00	2.22 (1.82–2.70)	1.60 (1.29–1.97)	2.55 (1.95–3.34)	1.50 (1.27–1.76)
	Adjusted	1.00	2.22 (1.82–2.70)	1.60 (1.29–1.97)	2.55 (1.95–3.34)	1.50 (1.27–1.76)
Fetal distress	Unadjusted	1.00	1.37 (0.99–1.91)	0.86 (0.59–1.26)	1.36 (0.84–2.20)	0.87 (0.66–1.15)
	Adjusted	1.00	1.42 (1.02–1.97)	0.89 (0.61–1.30)	1.41 (0.87–2.28)	0.90 (0.68–1.18)
APH	Unadjusted	1.00	1.23 (0.63–2.39)	0.98 (0.49–1.96)	1.42 (0.57–3.53)	1.31 (0.83–2.07)
	Adjusted	1.00	1.23 (0.63–2.38)	0.96 (0.48–1.92)	1.41 (0.57–3.51)	1.29 (0.82–2.04)
PPH	Unadjusted	1.00	0.96 (0.80–1.15)	0.98 (0.82–1.16)	1.06 (0.82–1.37)	1.01 (0.89–1.14)
	Adjusted	1.00	0.96 (0.80–1.15)	0.99 (0.83–1.17)	1.07 (0.82–1.38)	1.02 (0.90–1.15)
Cesarean delivery	Unadjusted	1.00	1.06 (0.95–1.18)	1.13 (1.02–1.25)	1.11 (0.95–1.30)	1.05 (0.97–1.13)
	Adjusted	1.00	1.06 (0.95–1.18)	1.13 (1.02–1.25)	1.11 (0.95–1.30)	1.05 (0.97–1.13)
Macrosomia	Unadjusted	1.00	1.71 (1.44–2.04)	1.30 (1.08–1.56)	1.59 (1.23–2.07)	1.27 (1.11–1.46)
	Adjusted	1.00	1.64 (1.37–1.95)	1.32 (1.10–1.58)	1.52 (1.16–1.97)	1.27 (1.11–1.46)
LBW	Unadjusted	1.00	2.01 (1.55–2.60)	1.69 (1.30–2.20)	2.40 (1.70–3.39)	1.51 (1.23–1.85)
	Adjusted	1.00	2.04 (1.58–2.65)	1.69 (1.30–2.20)	2.44 (1.73–3.45)	1.52 (1.23–1.86)
Neonatal jaundice	Unadjusted	1.00	1.21 (1.09–1.35)	3.41 (3.07–3.79)	1.15 (0.98–1.35)	2.06 (1.91–2.22)
	Adjusted	1.00	1.22 (1.10–1.36)	3.41 (3.07–3.79)	1.16 (0.99–1.36)	2.06 (1.92–2.22)
Data are presented as odds ratios (ORs) with 95% confidence intervals (CIs). Unadjusted and adjusted logistic regression analyses were employed to evaluate ORs, along with their 95% CIs. Adjusted models included age and body mass index as covariates. Subgroups within this table are defined based on individual diagnostic criteria frameworks and are not mutually exclusive. Abbreviations: APH, antepartum hemorrhage; GDM, gestational diabetes mellitus; IADPSG-only FPG, GDM women with only elevated fasting plasma glucose diagnosed by IADPSG; IADPSG-only post-load, GDM women with elevated only post-load hyperglycemia (1 h and/or 2 h) diagnosed by IADPSG; LBW, low birth weight; NGT, normal glucose tolerance; NICE-only FPG, GDM women with elevated only fasting plasma glucose diagnosed by NICE; NICE-only post-load, GDM women with elevated only post-load hyperglycemia (2 h) diagnosed by NICE; PPH, postpartum hemorrhage.						

To provide a clearer biological insight independent of diagnostic labels, we pooled the entire cohort (*N* = 19152) and re-categorized all participants into four strictly mutually exclusive groups based on their actual OGTT values: NGT (*n* = 13571, FPG < 5.1 and 1-h OGTT < 10.0 and 2-h OGTT < 7.8 mmol/L), FPG elevation (*n* = 1392, FPG ≥ 5.1 and 1-h OGTT < 10 and/or 2-h OGTT < 7.8 mmol/L), Post-load elevation (*n* = 3408, FPG < 5.1 and 1-h OGTT ≥ 10 and/or 2-h OGTT ≥ 7.8 mmol/L), and FPG + Post-load elevation (*n* = 781, FPG ≥ 5.1 and 1-h OGTT ≥ 10 and/or 2-h OGTT ≥ 7.8 mmol/L).

Intriguingly, data from these independent groups robustly support our conclusions. As shown in ***Supplementary Tables 4*** and ***5***, after adjusting for age and pre-pregnancy BMI, the FPG group exhibited substantially higher risks for gestational hypertension (adjusted OR = 2.59, 95% CI: 2.06–3.25) and preeclampsia (adjusted OR = 2.43, 95% CI: 1.93–3.06) than the Post-load group (OR = 1.29 and 1.27, respectively).

These findings further support the observation that fasting hyperglycemia is associated with higher risks of several adverse pregnancy outcomes. Because the IADPSG criteria lean heavily on the lower FPG threshold (≥ 5.1 mmol/L) while the NICE criteria identify a larger number of mild 2-h post-load elevations (≥ 7.8 mmol/L), the IADPSG framework successfully isolates a significantly higher-risk population, resulting in a lower GDM incidence but a higher complication rate compared to NICE (***Fig. 1*** and ***Table 5***).

## Discussion

To our knowledge, this is the first study directly comparing GDM prevalence using IADPSG and NICE criteria in China. Since adopting the IADPSG one-step approach in 2014 (updated in 2022), China has shifted from the ADA two-step method^[15–16]^. In our analysis of 19152 pregnant women (2015–2021), GDM prevalence was 20.39% by the IADPSG criteria. The NICE criteria classified 4533 women (23.67%) as having GDM, 627 more than the 3906 classified by IADPSG; however, 1675 women were uniquely identified by NICE, whereas 1048 were uniquely identified by IADPSG. These findings are consistent with the report from Vietnam (IADPSG criteria: 22.8%; NICE criteria: 24.2%, *n* = 2030)^[24]^ and the Hong Kong center of the HAPO study (majority Chinese, IADPSG criteria: 14.7%; NICE criteria: 17.4%, *n* = 1654)^[25]^, but inconsistent with data from India (IADPSG *vs.* NICE criteria: 25.10% *vs.* 11.60%, *n* = 680^[19]^; 23.80% *vs.* 21.50%, *n* = 353^[26]^), South Africa (25.10% *vs.* 17.00%, *n* = 554)^[27]^, Croatia (23.10% *vs.* 17.80%, *n* = 4646)^[28]^, Ireland (53.00% *vs.* 18.00%, *n* = 202)^[29]^, Finland (36.50% *vs.* 15.80%, *n* = 4939)^[30]^, Qatar (21.50% *vs.* 20.10%, *n* = 2000)^[18]^, and the other four centers of the HAPO study: Bellflower (IADPSG criteria: 25.50%; NICE criteria: 13.70%, *n* = 1981), Cleveland (25.00% *vs.* 16.80%, *n* = 797), Brisbane (12.40% *vs.* 11.80%, *n* = 1444), and Newcastle (15.30% *vs.* 13.90%, *n* = 668)^[25]^. These findings suggest significant heterogeneity in GDM prevalence trends across different diagnostic criteria when applied to diverse geographic regions and ethnic populations.

Furthermore, we assessed the agreement between the IADPSG and NICE criteria and demonstrated a κ statistic of 0.59 (moderate concordance), consistent with previous reports by Goyal *et al* (κ = 0.58)^[26]^. This modest agreement likely stems from NICE's omission of the 1-h glucose threshold and its adoption of a higher fasting but lower 2-h glucose cut-off. In our study, most GDM cases under the NICE criteria were diagnosed based on 2-h OGTT values (79.35% *vs.* 37.69% by IADPSG), whereas IADPSG identified more cases through FPG (39.25% *vs.* 14.67%). The resulting prevalence difference aligns with trends seen in an Argentine study comparing the Latin American Diabetes Association (ALAD) criteria (which uses standards similar to NICE) with the IADPSG criteria^[31]^. These findings suggest that differences in glycemic patterns and diagnostic thresholds across populations may influence GDM classification, and such variations may be mediated by distinct pathophysiological mechanisms. Therefore, to effectively reduce the adverse outcomes caused by GDM in different populations, further research is needed to explore whether personalized treatment strategies for specific populations are warranted.

The NICE criteria, developed based on cost-effectiveness considerations, may not offer a cost advantage in China because of the higher GDM prevalence they yield. Whether the NICE criteria can be applied to GDM diagnosis in China still requires further evaluation of pregnancy outcomes to evaluate their suitability. In our study, women in the GDM-I and GDM-N groups had significantly higher risks of adverse outcomes, including gestational hypertension, preeclampsia, preterm delivery, macrosomia, low birth weight, and neonatal jaundice, compared with those in the NGT group. However, the GDM-N group consistently showed lower incidences than the GDM-I group, with a significant reduction observed in neonatal jaundice. These data indicate that although the IADPSG criteria diagnose a lower prevalence of GDM than the NICE criteria, they identify a higher risk of adverse maternal and neonatal pregnancy outcomes.

Furthermore, we also analyzed the pregnancy outcomes of women who were diagnosed with GDM by NICE but not by IADPSG (GDM-NI). Compared with the NGT group, the GDM-NI group showed no significant increase in adverse outcomes, other than neonatal jaundice, consistent with prior reports^[19]^. However, when compared with the GDM-I group, the GDM-NI group had significantly lower rates of all adverse outcomes. This may be because GDM-NI cases were identified solely by elevated 2-h OGTT values, which are associated with lower risks than those identified by fasting hyperglycemia (***Table 6***)^[22–23]^. These results indicate that the GDM-NI group had outcomes similar to NGT but better than GDM-I. However, as the GDM-NI group received no treatment while the GDM-I group did, this comparison reflects the combined effects of diagnostic classification and subsequent clinical management rather than the intrinsic diagnostic performance of the two criteria alone. Therefore, caution is warranted in concluding that the NICE criteria are less suitable for Chinese pregnant women based solely on these untreated outcomes. It is noteworthy that the increased risks of adverse outcomes associated with GDM diagnosed by IADPSG criteria remained significant after adjustment for maternal age and BMI at OGTT, supporting the robustness of our findings. The attenuation of ORs after adjustment suggests that maternal age and BMI may partly confound the observed associations, but do not fully explain the association between IADPSG-defined GDM and adverse pregnancy outcomes, because other variables were unavailable, such as parity, previous GDM, family history of diabetes, gestational weight gain, smoking, *in vitro* fertilization, and assisted reproduction.

Given participant overlap between the GDM-I and GDM-N groups, we reclassified the cohort into four mutually exclusive subgroups: NGT, IADPSG^+^/NICE^−^ (GDM-IN), IADPSG^+^/NICE^+^ (GDM-All), and GDM-NI. As shown in ***Table 4*** and ***Supplementary Table 2***, compared with the NGT group, the incidence ranking of most adverse pregnancy outcomes was highly consistent. Specifically, the rates of preeclampsia, preterm delivery, and macrosomia followed the order: GDM-IN > GDM-I > GDM-All > GDM-N > GDM-NI. This trend is similar to several indicators reported by Meek *et al*^[32]^, underscoring that elevated FPG is independently associated with increased risk of these adverse pregnancy outcomes and affirming the reliability of our data.

Notably, despite glucose management, the GDM-I group had worse pregnancy outcomes compared with women classified as GDM-NI, underscoring the difficulty of improving outcomes (*e.g.*, macrosomia, cesarean rates) through glycemic control alone^[33–34]^. Future studies are warranted to evaluate innovative treatment strategies that address the multifactorial nature of GDM and its impact on maternal and neonatal health.

Several limitations of this study should be acknowledged. First, the findings of this study do not reflect the entire population of China as the study was undertaken only at a tertiary hospital in Nanjing. Second, several maternal and neonatal outcomes, such as small for gestational age (SGA)/large for gestational age (LGA) and neonatal hypoglycemia, were excluded due to insufficient or incomplete data. For example, some pregnant women lacked early ultrasound confirmation, which is critical for accurately defining SGA or LGA based on population standards. Third, due to the retrospective nature of the electronic medical registry database, several well-known confounding factors—such as parity, family history of diabetes, gestational weight gain, smoking habits, *in vitro* fertilization, and assisted reproduction—were either unavailable or had substantial missing data. Although we adjusted for core maternal characteristics (age and pre-pregnancy BMI) and strictly excluded individuals with pre-existing chronic conditions like pre-pregnancy hypertension and diabetes, the possibility of residual confounding cannot be entirely eliminated. Consequently, these unmeasured factors might influence the exact magnitude of the observed risks. Fourth, this study evaluated the NICE diagnostic thresholds rather than its full screening strategy. Because our cohort underwent universal OGTT screening rather than the risk-factor-based selective testing recommended by NICE, the GDM incidence and risk estimates might differ if the complete NICE screening workflow were applied. Fifth, future studies should assess postpartum outcomes in women negative by IADPSG criteria but positive by NICE criteria, particularly given evidence linking discordant GDM diagnoses to later cardiometabolic risk^[35]^. Because all women diagnosed by the IADPSG criteria received treatment whereas women diagnosed only by the NICE criteria did not, differences in pregnancy outcomes may partly reflect treatment effects in addition to differences in diagnostic classification. Finally, information on GDM treatment was not collected, which may affect the adverse outcomes for some pregnant women.

Because women diagnosed according to the IADPSG criteria received clinical intervention whereas those classified as GDM-NI did not, the present findings should be interpreted as reflecting the combined effects of diagnostic classification and subsequent clinical management rather than the diagnostic criteria alone. Future prospective studies applying both diagnostic criteria before treatment allocation are warranted.

In conclusion, this retrospective cohort study of 19152 pregnant Chinese women demonstrates that within the current clinical management setting, women identified by the IADPSG criteria had higher observed risks of several adverse pregnancy outcomes than women identified exclusively by the NICE criteria. However, because diagnostic classification determined treatment, these findings cannot establish the comparative predictive superiority of the two criteria.

## Additional information

The online version contains supplementary materials available at http://www.jbr-pub.org.cn/article/doi/10.7555/JBR.39.20250350.
