# Supplementary material for: Maternal and neonatal outcomes among Chinese women meeting NICE but not IADPSG criteria for gestational diabetes mellitus: A retrospective cohort study
Source: J Biomed Res. 2026 Jul 28;40(4):436–47. doi: 10.7555/JBR.39.20250350 (PMC13438299; doi:10.7555/JBR.39.20250350)
Supplement: Supplementary file 1 [file jbr-40-4-436-S1.pdf]

Available online at www.jbr-pub.org.cn

Open Access at PubMed Central

Journal of Biomedical Research, 2026 40(4): 1–3

Supplementary Data

JBR

Maternal and neonatal outcomes among Chinese women meeting NICE but not IADPSG criteria for gestational diabetes mellitus: A retrospective cohort study

Haijuan Yu[1,2,3], Yuan Qin[4], Runlian Tao[4], Yuanyuan Chen[4,✉], Li Yuan[4,✉], Lizhou Sun[1,✉]

[1]*Department of Obstetrics, the First Affiliated Hospital of Nanjing Medical University, Nanjing, Jiangsu 210029, China;*
[2]*Department of Obstetrics, Nanjing Jiangning Hospital of Chinese Medicine, Nanjing, Jiangsu 211100, China;*
[3]*Affiliated Jiangning Hospital of Chinese Medicine, China Pharmaceutical University, Nanjing, Jiangsu 211198, China;*
[4]*Department of Biochemistry and Molecular Biology, Jiangsu Key Laboratory of Molecular Targets and Intervention for Metabolic Diseases, Nanjing Medical University, Nanjing, Jiangsu 211166, China.*

Supplementary Table 1 **Maternal and neonatal outcomes by OGTT diagnostic category**

	Plasma glucose level (mmol/L)			Diagnostic criteria
	FPG	1-h OGTT	2-h OGTT	
IADPSG	⩾ 5.1	⩾ 10.0	⩾ 8.5	If any value meets the requirements, it is diagnosed as GDM
NICE	⩾ 5.6	–	⩾ 7.8	

To convert glucose value from mmol/L to mg/dL, multiply by 18. "–" indicates data not available.
Abbreviations: FPG, fasting plasma glucose level; IADPSG, International Association of Diabetes and Pregnancy Study Groups; NICE, National Institute for Health and Care Excellence; OGTT, oral glucose tolerance test.

✉Corresponding authors: Yuanyuan Chen, E-mail: yuanyuanch@njmu.edu.cn, ORCID: 0000-0003-4379-6267; Li Yuan, E-mail: yuanli@njmu.edu.cn, ORCID: 0000-0002-0284-5854; Lizhou Sun, E-mail: lizhou_sun@163.com, ORCID: 0000-0002-0465-8825.

Received: 23 August 2025; Revised: 26 November 2025; Accepted: 01 December 2025; Available online: 12 December 2025; Published date: 28 July 2026

CLC number: R714.256, Document code: A

The authors reported no conflicts of interest.

This is an open access article under the Creative Commons Attribution (CC BY 4.0) license, which permits others to distribute, remix, adapt and build upon this work, for commercial use, provided the original work is properly cited.

© 2026 by Journal of Biomedical Research.

https://doi.org/10.7555/JBR.39.20250350

Supplementary Table 2 **Maternal and neonatal outcomes by OGTT diagnostic category**

Outcomes	IADPSG^{-}/NICE^{-} (NGT)	IADPSG^{+}/NICE^{-} (GDM-IN)	IADPSG^{+} /NICE^{+} (GDM-All)	IADPSG^{-}/NICE^{+} (GDM-NI)	*P*
n	13 571	1 048	2 858	1 675	
Gestational hypertension [*n* (%)]	354 (2.61)^{A}	49 (4.68)^{BC}	155 (5.42)^{C}	53 (3.16)^{AB}	< 0.001
Preeclampsia [*n* (%)]	390 (2.87)^{A}	75 (7.16)^{B}	147 (5.14)^{BC}	61 (3.64)^{AC}	< 0.001
Preterm delivery [*n* (%)]	553 (4.07)^{A}	83 (7.92)^{B}	226 (7.91)^{B}	82 (4.9)^{A}	< 0.001
Fetal distress [*n* (%)]	273 (2.01)^{A}	29 (2.77)^{A}	58 (2.03)^{A}	31 (1.85)^{A}	0.371
APH [*n* (%)]	72 (0.53)^{A}	7 (0.67)^{A}	19 (0.66)^{A}	14 (0.84)^{A}	0.400
PPH [*n* (%)]	1 314 (9.68)^{A}	92 (8.78)^{A}	283 (9.90)^{A}	167 (9.97)^{A}	0.731
Cesarean delivery [*n* (%)]	5 204 (38.35)^{A}	421 (40.17)^{AB}	1 174 (41.08)^{B}	637 (38.03)^{AB}	0.034
Macrosomia [*n* (%)]	893 (6.58)^{A}	121 (11.55)^{B}	280 (9.8)^{BC}	127 (7.58)^{AC}	< 0.001
LBW [*n* (%)]	334 (2.46)^{A}	48 (4.58)^{BC}	135 (4.72)^{C}	50 (2.99)^{AB}	< 0.001
Neonatal jaundice [*n* (%)]	4 890 (36.03)^{A}	482 (45.99)^{B}	1 671 (58.47)^{C}	681 (40.66)^{D}	< 0.001

Data are expressed as numbers (percentages). Categorical variables were compared using the chi-square test with Bonferroni's post hoc test. Values in the same row with different superscript letters differ significantly ($P < 0.05$), and a value with superscripts 'AB' is not significantly different from values with superscript 'A' or 'B'.
Abbreviations: APH, antepartum hemorrhage; GDM-All, diagnosed with GDM based on IADPSG criteria and NICE criteria (IADPSG^{+}/NICE^{+}); GDM-IN, diagnosed with GDM based on IADPSG criteria but not NICE criteria (IADPSG^{+}/NICE^{-}); GDM-NI, diagnosed with GDM based on NICE criteria but not IADPSG criteria (NICE^{+}/IADPSG^{-}); LBW, low birth weight; NGT, normal glucose tolerance; OGTT, oral glucose tolerance test; PPH, postpartum hemorrhage.

Supplementary Table 3 **Odds ratios for adverse pregnancy outcomes by OGTT diagnostic category**

Outcomes	OR type	IADPSG^{-}/NICE^{-} (NGT)	IADPSG^{+}/NICE^{-} (GDM-IN)	IADPSG^{+} /NICE^{+} (GDM-All)	IADPSG^{-}/NICE^{+} (GDM-NI)
n		13 571	1 048	2 858	1 675
Gestational hypertension	Unadjusted	1.00 (Ref.)	1.83 (1.35–2.49)	2.14 (1.77–2.60)	1.22 (0.91–1.64)
	Adjusted	1.00	1.70 (1.25–2.31)	2.05 (1.68–2.49)	1.17 (0.87–1.58)
Preeclampsia	Unadjusted	1.00	2.61 (2.03–3.36)	1.83 (1.51–2.23)	1.28 (0.97–1.68)
	Adjusted	1.00	2.44 (1.88–3.16)	1.77 (1.46–2.16)	1.24 (0.94–1.63)
Preterm delivery	Unadjusted	1.00	2.03 (1.59–2.57)	2.02 (1.72–2.37)	1.21 (0.96–1.54)
	Adjusted	1.00	2.03 (1.59–2.58)	2.03 (1.73–2.38)	1.21 (0.96–1.54)
Fetal distress	Unadjusted	1.00	1.39 (0.94–2.04)	1.01 (0.76–1.34)	0.92 (0.63–1.34)
	Adjusted	1.00	1.42 (0.96–2.10)	1.05 (0.79–1.40)	0.94 (0.65–1.37)
APH	Unadjusted	1.00	1.26 (0.58–2.75)	1.26 (0.76–2.09)	1.58 (0.89–2.81)
	Adjusted	1.00	1.26 (0.58–2.75)	1.23 (0.74–2.05)	1.57 (0.88–2.79)
PPH	Unadjusted	1.00	0.90 (0.72–1.12)	1.03 (0.90–1.17)	1.03 (0.87–1.22)
	Adjusted	1.00	0.90 (0.72–1.12)	1.03 (0.90–1.18)	1.04 (0.86–1.23)
Cesarean delivery	Unadjusted	1.00	1.08 (0.95–1.23)	1.12 (1.03–1.22)	0.99 (0.89–1.10)
	Adjusted	1.00	1.08 (0.95–1.23)	1.12 (1.03–1.22)	0.99 (0.89–1.10)
Macrosomia	Unadjusted	1.00	1.85 (1.52–2.27)	1.54 (1.34–1.78)	1.17 (0.96–1.41)
	Adjusted	1.00	1.78 (1.45–2.17)	1.52 (1.32–1.76)	1.15 (0.95–1.40)
LBW	Unadjusted	1.00	1.90 (1.40–2.59)	1.97 (1.60–2.41)	1.22 (0.90–1.65)
	Adjusted	1.00	1.93 (1.42–2.63)	1.98 (1.62–2.43)	1.23 (0.91–1.66)
Neonatal jaundice	Unadjusted	1.00	1.51 (1.33–1.72)	2.50 (2.30–2.71)	1.22 (1.10–1.35)
	Adjusted	1.00	1.53 (1.35–1.74)	2.52 (2.32–2.73)	1.22 (1.10–1.36)

Data are presented as odds ratios (ORs) with 95% confidence intervals (CIs). Unadjusted and adjusted logistic regression analyses were employed to evaluate ORs, along with their 95% CIs. Adjusted models included age and pre-pregnancy body mass index as covariates.
Abbreviations: APH, antepartum hemorrhage; GDM-All, diagnosed with GDM based on IADPSG criteria and NICE criteria (IADPSG^{+}/NICE^{+}); GDM-IN, diagnosed with GDM based on IADPSG criteria but not NICE criteria (IADPSG^{+}/NICE^{-}); GDM-NI, diagnosed with GDM based on NICE criteria but not IADPSG criteria (NICE^{+}/IADPSG^{-}); LBW, low birth weight; NGT, normal glucose tolerance; PPH, postpartum hemorrhage; Ref., reference.

Supplementary Table 4 **Association between fasting/post-load glucose levels and pregnancy outcomes**

Outcomes	NGT (n = 13571)	FPG (n = 1392)	Post-load (n = 3408)	FPG + Post-load (n = 781)	P
Gestational hypertension	354 (2.61)^{A}	64 (4.60)^{B}	118 (3.46)^{B}	75 (9.60)^{C}	< 0.001
Preeclampsia	390 (2.87)^{A}	99 (7.11)^{B}	125 (3.67)^{A}	59 (7.55)^{B}	< 0.001
Preterm delivery	553 (4.07)^{A}	127 (9.12)^{B}	192 (5.63)^{C}	72 (9.22)^{B}	< 0.001
Fetal distress	273 (2.01)	41 (2.95)	61 (1.79)	16 (2.05)	0.077
APH	72 (0.53)	10 (0.72)	23 (0.67)	7 (0.90)	0.324
PPH	1314 (9.68)	131 (9.41)	331 (9.71)	80 (10.24)	0.940
Cesarean delivery	5204 (38.35)	559 (40.16)	1352 (39.67)	272 (41.10)	0.166
Macrosomia	893 (6.58)^{A}	150 (10.78)^{B}	272 (7.98)^{C}	321 (13.57)^{B}	< 0.001
LBW	334 (2.46)^{A}	70 (5.03)^{B}	121 (3.55)^{B}	42 (5.38)^{B}	< 0.001
Neonatal jaundice	4890 (36.03)^{A}	560 (40.23)^{B}	1821 (53.43)^{C}	453 (58.00)^{C}	< 0.001

Data are expressed as numbers (percentages). Categorical variables were compared using the chi-square test with Bonferroni's post hoc test. Values in the same row with different superscript letters differ significantly ($P < 0.05$), and a value with superscripts 'AB' is not significantly different from values with superscript 'A' or 'B'.
Abbreviations: APH, antepartum hemorrhage; FPG + Post-load elevation (FPG ⩾ 5.1 and 1-h OGTT ⩾ 10 and/or 2-h OGTT ⩾ 7.8 mmol/L); LBW, low birth weight; NGT, normal glucose tolerance (FPG < 5.1 and 1-h OGTT < 10 and 2-h OGTT < 7.8 mmol/L); OGTT, oral glucose tolerance test; FPG elevation (FPG ⩾ 5.1 and 1-h OGTT < 10 and/or 2-h OGTT < 7.8 mmol/L); Post-load elevation (FPG < 5.1 and 1-h OGTT ⩾ 10 and/or 2-h OGTT ⩾ 7.8 mmol/L); PPH, postpartum hemorrhage.

Supplementary Table 5 **Odds ratios for adverse pregnancy outcomes by fasting/post-load glucose levels**

Outcomes	OR type	NGT (n = 13 571)	FPG (n = 1 392)	Post-load (n = 3 408)	FPG + Post-load (n = 781)
Gestational hypertension	Unadjusted	1.00 (Ref.)	1.80 (1.37–2.36)	1.34 (1.08–1.66)	3.97 (3.06–5.15)
	Adjusted	1.00	2.59 (2.06–3.25)	1.29 (1.05–1.58)	2.76(2.08–3.67)
Preeclampsia	Unadjusted	1.00	2.59 (2.06–3.25)	1.29 (1.05–1.58)	2.76 (2.08–3.67)
	Adjusted	1.00	2.43 (1.93–3.06)	1.27 (1.04–1.57)	2.47 (1.86–3.30)
Preterm delivery	Unadjusted	1.00	2.36 (1.93–2.89)	1.41 (1.19–1.66)	2.39 (1.85–3.09)
	Adjusted	1.00	2.37 (1.94–2.90)	1.41 (1.19–1.67)	2.40 (1.86–3.11)
Fetal distress	Unadjusted	1.00	1.49 (1.06–2.06)	0.89 (0.67–1.18)	1.02 (0.61–1.70)
	Adjusted	1.00	1.52 (1.09–2.12)	0.91 (0.69–1.21)	1.08 (0.65–1.80)
APH	Unadjusted	1.00	1.35 (0.70–2.63)	1.26 (0.78–2.01)	1.67 (0.77–3.66)
	Adjusted	1.00	1.35 (0.70–2.63)	1.26 (0.78–2.01)	1.67 (0.77–3.66)
PPH	Unadjusted	1.00	0.97 (0.80–1.17)	1.00 (0.88–1.14)	1.07 (0.84–1.35)
	Adjusted	1.00	0.97 (0.80–1.17)	1.01 (0.88–1.14)	1.07 (0.84–1.36)
Cesarean delivery	Unadjusted	1.00	1.08 (0.97–1.21)	1.06 (0.98–1.14)	1.12 (0.97–1.30)
	Adjusted	1.00	1.08 (0.97–1.21)	1.06 (0.98–1.14)	1.12 (0.97–1.30)
Macrosomia	Unadjusted	1.00	1.72 (1.43–2.06)	1.23 (1.07–1.42)	2.23 (1.80–2.77)
	Adjusted	1.00	1.65 (1.37–1.98)	1.23 (1.07–1.42)	2.11 (1.70–2.62)
LBW	Unadjusted	1.00	2.10 (1.61–2.73)	1.46 (1.18–1.80)	2.25 (1.62–3.13)
	Adjusted	1.00	2.13 (1.64–2.77)	1.47 (1.19–1.81)	2.31 (1.66–3.21)
Neonatal jaundice	Unadjusted	1.00	1.20 (1.07–1.34)	2.04 (1.89–2.20)	2.45 (2.12–2.84)
	Adjusted	1.00	1.20 (1.07–1.34)	2.04 (1.89–2.20)	2.45 (2.12–2.84)

Data are OR (95% CI). Unadjusted and adjusted logistic regression analyses were employed to evaluate odds ratios (ORs), along with their 95% confidence intervals (CIs). Adjusted models included age and pre-pregnancy body mass index as covariates.
Abbreviations: APH, antepartum hemorrhage; FPG + Post-load elevation (FPG ⩾ 5.1 and 1-h OGTT ⩾ 10 and/or 2-h OGTT ⩾ 7.8 mmol/L); LBW, low birth weight; NGT, normal glucose tolerance (FPG < 5.1 and 1-h OGTT < 10 and 2-h OGTT < 7.8 mmol/L); OGTT, oral glucose tolerance test; FPG elevation (FPG ⩾ 5.1 and 1-h OGTT < 10 and/or 2-h OGTT < 7.8 mmol/L); Post-load elevation (FPG < 5.1 and 1-h OGTT ⩾ 10 and/or 2-h OGTT ⩾ 7.8 mmol/L); PPH, postpartum hemorrhage; Ref., reference.
